# Why Seed Physiology Is Important for Genebanking

**DOI:** 10.3390/plants9050584

**Published:** 2020-05-02

**Authors:** Katherine J. Whitehouse, Fiona R. Hay, Charlotte Lusty

**Affiliations:** 1 Australian Grains Genebank, Agriculture Victoria Research, Departments of Jobs, Precincts and Regions, Private Bag 260, Horsham, Victoria 3401, Australia; katherine.whitehouse@agriculture.vic.gov.au; 2 Department of Agroecology, Aarhus University, Forsøgsvej 1, 4200 Slagelse, Denmark; 3 Global Crop Diversity Trust, Platz Der Vereinten Nationen 7, 53113 Bonn, Germany; charlotte.lusty@croptrust.org

**Keywords:** agrobiodiversity, genebank, genebank management, plant genetic resources, seed physiology, seed quality management

## Abstract

Genebank management is a field in its own right; it is multifaceted, requiring a diverse set of skills and knowledge. Seed physiology is one area that is critical to the successful operation of seed genebanks, requiring understanding of seed quality during development and maturation, seed dormancy and germination, and seed longevity in storage of the target species. Careful management of the workflow between these activities, as seeds move from harvest to storage, and the recording and management of all relevant associated data, is key to ensuring the effective conservation of plant genetic resources. This review will discuss various aspects of seed physiology that genebank managers should be aware of, to ensure appropriate decisions are made about the handling and management of their seed collections.

## 1. Introduction

Storing seeds in genebanks is the most effective way of conserving and sharing most of our existing agrobiodiversity (that of orthodox species). It is also a relatively simple activity; seeds are dried and stored at low temperature. However, within that simple statement lies a whole series of operations, which, if not carefully followed and controlled, risk the loss of the agrobiodiversity that genebanks are meant to be conserving. Effective management of the individual operations and of the whole workflow, especially when managing a crop genebank with thousands of accessions, requires some understanding of seeds and how they might be impacted in terms of their quality and viability during the different production and processing steps. In this review, we discuss various aspects of seed physiology that we consider particularly relevant to genebanks, including understanding whether a seed is viable or just dormant and how dormancy might be overcome; the acquisition and loss of the ability to tolerate desiccation during seed development; seed ageing leading to loss of viability; understanding how long seed lots will remain above a threshold viability level based on genebank viability monitoring data, modelling, and comparative longevity experiments; how seed longevity might be affected by various pre- and post-harvest factors; and the statistics of analysing seed germination/viability data. The review is directed primarily towards genebanks that conserve agrobiodiversity; the science of seed banking to conserve species diversity has been reviewed elsewhere [1,2]. Some wild species seed banks invest significant resources into researching the seeds of the species they conserve. In contrast, many agricultural genebanks do not have a mandate to do much research and, in the interests of efficiency in genebank operations, avoid collecting data that could be classified as non-essential to managing their collections. We nonetheless touch upon various areas where genebanks could collect data that would be of interest and use to the network of genebanks and to the scientific community. In relation to this, we also encourage renewed interaction between genebanks and the seed science and testing community.

## 2. Understanding Why a Seed Lot Is Showing No or Low Germination

Air-dry seeds of some orthodox species are predicted to survive for very long periods at sub-zero temperatures, typical of long-term genebank storage conditions (−20 °C) [3]. However, many genebanks regenerate [regeneration is used to describe the process of planting seeds to produce more seeds for genebank storage, be that as a result of declining viability, where the process might also be called ‘rejuvenation’, or to increase the number of seeds available (‘multiplication’)] their material more frequently than longevity predictions would suggest is necessary (unpublished data). This may be because insufficient seeds are being placed into storage [4], because seeds are being distributed at higher rates than expected or a viability monitoring result indicates low viability (i.e., below the acceptable threshold), or simply for reassurance that the seeds in storage, because they are ‘fresh’, must be viable. Indeed, some accessions may be regenerated frequently because it is difficult to produce seeds in large quantities in one season (because of, for example, a small plot size, poor plant establishment, or low fecundity) and/or because the initial germination result is poor. There are two fundamental reasons why a seed lot might show no or low germination, either in the initial germination test or in a viability monitoring germination test, including seed dormancy and/or non-optimal germination conditions or low seed viability. Indeed, the most important first consideration is to understand whether or not the result is an accurate reflection of the viability of the seed lot.

### 2.1. Viability versus Germinability

Ultimately, it is only when a seed germinates that we can be sure that the genetic information it contains can be expressed or exploited, be that for crop production or for scientific research. Thus, a germination test is considered the ultimate method for monitoring ‘viability’, even though it requires knowledge of dormancy-breaking treatments and optimum germination conditions. However, there are other ways in which the viability of a seed lot can be assessed. At the end of a germination test, any seeds that have not germinated could be assessed via a ‘cut-test’ to see if they are dead seeds (soft, mouldy); seeds that have not taken up moisture (hard-seededness, a trait that is particular prevalent in seeds from species in families such as the Fabaceae and Malvaceae); empty seeds; or seeds that still have firm, normal-coloured tissues. Other viability tests would be made on a separate sample of seeds. The International Seed Testing Association has a whole chapter of their *International Rules for Seed Testing* dedicated to the use of the tetrazolium test to rapidly assess seed lot viability, based on the living parts of the viable seed staining red, with optimized procedures for specific species or genera. The method usually involves imbibition of water, seed sectioning, and exposure to a solution of 2,3,5-triphenyl tetrazolium chloride or bromide for a specified period of time [5]. How to interpret the results (staining pattern) is also described [5]. The tetrazolium test is often criticised for being somewhat subjective; therefore, it should only be used instead of a germination test when it is not possible to remove seed dormancy. The species and genera included in the *International Rules for Seed Testing* tetrazolium chapter [5] cover the most important crops and wild species conserved by genebanks. Fluorescein diacetate has also been used as a vital stain for seeds, in particular for orchid seeds, which are very small and would thus be difficult to evaluate using tetrazolium [6,7], but also for larger seeds [8]. The methodology is similar to the tetrazolium test, except viability is assessed by observation under a fluorescence microscope, making this method less attractive than the tetrazolium test, which generally does not require any specialised equipment.

If the viability test result is consistent with the germination result, the two test results together can be considered reliable. Indeed, this is how the tetrazolium viability test is used in some genebanks, particularly those working on wild species, where the dormancy mechanism of different and diverse species may not always be known. When the two results are comparable, then the dormancy-breaking treatment(s) and/or germination test conditions are considered appropriate. If the viability result is higher than the germination result, further dormancy-breaking treatment(s) and/or germination conditions may be tried, if there are sufficient seeds available. It is also possible that the viability result is less than the germination result, in which case the tetrazolium test conditions may not be optimal. Some genebanks systematically use the tetrazolium test for viability monitoring, perhaps because it can ultimately be more straightforward than lots of different, multi-step germination tests. This is not recommended, except perhaps under special circumstances.

### 2.2. Seed Dormancy and Germination Behaviour

A non-dormant seed will readily germinate given appropriate environmental conditions, most importantly, moisture and temperature. The optimum temperature range for germination can vary between species, but is likely to reflect the ambient temperature in the habitat where the species naturally occurs or is typically planted, and at the time of year when the plant is expected to germinate [9,10]. In contrast, dormancy mechanisms can be multifaceted and diverse, although still climate-related [9,10]. Species-specific information on germination protocols and dormancy breaking treatments are widely available, again particularly for crop species and their wild relatives [5,10,11,12,13]. It is, however, important to be aware that, in some species, dormancy may be released or induced during storage, which may mean that the germination procedure initially used may have to be adapted following storage [14,15,16].

Where dormancy is suspected, because a cut test indicates that seeds are probably still viable at the end of a germination test, further germination tests with different dormancy-breaking treatments should be performed. The same literature as described in the previous section will help guide what treatments should be prioritized for testing, for example, if the ‘problem’ species is closely related (same genus) and comes from a similar environment as a species for which dormancy behaviour is documented. Complex, multi-factorial experiments are unlikely to be conducted in genebank laboratories (Table 1), unless there is a plan to publish the findings, in which case a more structured experimental design that can be appropriately analysed (see Section 4 below) might be better received. Nonetheless, it is helpful to share any such ‘new’ data on the germination requirements of previously unstudied species—precisely because it is useful information for other genebanks and for end-users. Indeed, most genebanks give germination advice when they distribute seed samples, if the information is available.

### 2.3. Desiccation Tolerance

Not all plant species produce seeds that tolerate the drying procedure, which is one of the steps in preparing seeds for genebank storage, and seeds of some other species, even if desiccation-tolerant, do not survive for long periods under low-temperature storage. However, the proportion of species that show this non-orthodox seed storage behaviour is low (8%, [17]), and of course, species that are already effectively managed and conserved in genebanks are clearly orthodox. However, seeds of orthodox species can be desiccation-intolerant if they are harvested too early or, conversely, if germination has commenced, for example, if the seeds are prone to vivipary. Seeds acquire physiological traits, including desiccation tolerance, during development. The relative timing of the acquisition of desiccation tolerance varies between species, but is normally acquired by or around the time that seeds reach mass maturity, when the seeds stop accumulating dry matter, and thus reach their maximum dry weight ([18] and references therein). Because dry weight will be reflected in the fresh weight of the seeds and other physical characteristics of the seed, it is perhaps unlikely that seeds of an orthodox species will be harvested before desiccation tolerance is acquired, unless the species flowers and fruits asynchronously. For species with such indeterminate flowering, it may be difficult to avoid harvesting seeds that have yet to acquire desiccation tolerance, and this may be a reason that a seed lot shows poor germination in the initial viability test. It may be possible to remove the desiccation intolerant seeds in the seed lot, by sorting based on density, size, and/or appearance. Alternatively, slow drying the whole seed lot or pre-sorting the seeds according to likely maturity and then slow drying the least mature seeds may allow maturation events to continue in the least mature seeds [19].

Vivipary is a characteristic of some species or varieties whose seeds have little or no dormancy as the seeds mature on the plant. If the seeds are exposed to germination-promoting conditions, in particular, high moisture owing to, for example, late rain, then the seeds may start to germinate while still on the plant [20]. Vivipary is common in tomatoes and peppers, as well as some cereals (e.g., temperate rice). Upon germination, orthodox seeds lose the ability to tolerate desiccation. Imbibition of water alters the physical properties of the membrane lipid bilayer, making the cellular membranes susceptible to injury upon subsequent desiccation [21,22,23]. Furthermore, as metabolism is reinstated upon imbibition, subsequent desiccation can lead to the initiation of further lipid peroxidation (see below) in the dehydrated tissues, which can continue upon re-imbibition [21]. Hence, any seeds showing on-plant germination will have to be discarded; indeed, it may not be possible to identify all the seeds that have started to germinate and thus lost desiccation tolerance, meaning that the entire seed lot may have to be rejected.

### 2.4. Seed Ageing

Seed ageing is inevitable, regardless of the conditions under which the seeds are stored, though of course, the aim of genebanking is to slow down the rate of ageing so as to preserve the genetic integrity of the seeds and to make sure the genetic information is available for use. Seeds are hygroscopic, meaning they exchange water with their surroundings until they reach equilibrium. The tendency of water to move into the tissues from the external environment is dependent upon the relative humidity (RH) of the atmosphere and the moisture content of the seed. It also depends on the chemical composition (oil content), size, and seed coat properties. Seeds with a higher oil content have a lower moisture content at a specific RH compared with lower oil content seeds. How a seed interacts with water can be explained by sorption isotherms, the shape of which reflects the availability of water within the seeds to support different chemical reactions [24,25,26,27]. After the attainment of mass maturity, that is, the end of seed-filling when seeds have reached maximum dry weight, seeds equilibrate with the ambient environmental conditions, usually by losing moisture, unless conditions are very humid. Nonetheless, they may still be harvested at a moisture content conducive to high rates of ageing reactions. This is why it is critical that seeds are dried as quickly as possible following harvest, in order to reduce the rate of ageing reactions (unless slow drying may allow the continuation of the acquisition of desiccation tolerance, as discussed above).

The type and rate of ageing reactions depend on seed moisture content, temperature, and oxygen availability [28]. For example, respiration will only occur in the presence of oxygen and at a high enough RH that the cytoplasm allows molecular motion. As a result, seeds can be classified as either undergoing wet or dry ageing, determined by the viscosity of the cytoplasm (low viscosity at RH ≥ca.75% (wet ageing) and highly viscous/glassy state at RH <ca.75% (dry ageing)). The difference in the types of reactions that occur during these two states differentially impacts hormonal regulation and signalling pathways involved in longevity [29]. The major contributors to seed deterioration, resulting in loss of membrane integrity, reduced energy metabolism, protein carbonylation, impairment in RNA and protein synthesis, and RNA degradation, are lipid peroxidation and free radical accumulation [30]. DNA damage inhibits effective transcription and replication [31], key processes that are activated during the imbibition stage of germination [32,33]. In this stage, repair mechanisms are activated, in order to restore a functional physiological state [32,33]. However, if damage has accumulated above a certain threshold, seeds will begin to show a delay in germination, as it takes longer to repair all the damage. This explains why seed lot vigour declines and the proportion of abnormal seedlings increases, before complete loss of viability [20], although the temporal pattern from the origin of deterioration to death, either within an individual seed or a seed lot, is still not fully understood [29]. It is important to note however, that if vigour and/or abnormal germination was used to indicate the extent to which a seed lot has already aged, there may be an expectation that accessions be regenerated at an earlier stage of the ageing process. This could lead to more frequent regeneration, which would not be encouraged owing to the inherent costs and risks. Thus, better understanding of the vigour of accessions and how it changes during storage is again perhaps something that is more desirable from a scientific perspective and/or of interest to end-users, rather than being something that genebanks should aspire to assess routinely (Table 1).

## 3. Understanding How Long a Seed Lot Will Continue to Show Good Germination and the Factors That Influence That Period

Thresholds for the acceptable percentage viability of a seed lot have been defined in various standards [34,35]. Understanding how long a seed lot is likely to maintain viability above the threshold value will improve the efficiency of genebank operations. For example, in some cases, monitoring intervals could be extended beyond 5 or 10 years, which are the ‘default’ intervals for species for which evidence of seed longevity is lacking (but which are nonetheless ‘expected’ to be short- or long-lived, respectively, in long-term genebank storage, at −18 °C, in hermetically closed containers [34]). This will save use of seeds, resources, and particularly staff time. Conversely, expectations regarding seed longevity may also highlight where there might be problems owing to biology, perhaps, or where interventions might be appropriate in the seed harvesting–processing–storage chain.

### 3.1. Historical Viability Monitoring Data

A number of genebanks have now published historical viability monitoring data for seeds in medium- and/or long-term storage, including the United States Department of Agriculture [36]; the National Agrobiodiversity Center of South Korea [37]; the Centre for Genetic Resources, the Netherlands (CGN [38,39]); the International Rice Research Institute (IRRI [40]); and the International Livestock Research Institute (ILRI [41,42]). Some of these publications highlight the problems of analysing such data, including changes in protocols and/or storage conditions, failure to overcome dormancy, and censoring. Testing of seed lots in genebank storage often ceases once the seed lot is ‘replaced’ by newly harvested seeds following a cycle of regeneration (literally or metaphorically in that a seed lot may simply cease to be actively managed; Table 1). Thus, the data are censored and most of the data collected have values between 100% and whatever the viability standard might be, typically 75% or 85%, and higher if seed lots are primarily replaced owing to low seed quantity. This makes the data difficult to analyse using methods such as generalized linear modelling (e.g., probit analysis) of germination percentage data, which might otherwise be used (see below).

Nonetheless, these papers in general both confirm the effectiveness of seed genebanks as a means of conserving agrobiodiversity, and perhaps flag accessions that are likely to have shorter seed longevity, for example, accessions of carrot, parsnip, and onion [36] or temperate varieties of rice [40], or accessions that show unexpected behaviour in response to genebank storage, as reported for wheat and barley [39]. In the case of the analysis of the data from the IRRI genebank, it was not deemed necessary to change the monitoring interval, largely because there appeared to be a trend of declining longevity of the seeds being placed into storage, for seeds harvested between 1992 and 2001 (the last year’s harvest covered by the analysis was 2002 [40]). On the other hand, CGN concluded that they could delay the first monitoring test to 25 years after harvest (seeds in long-term storage at −20 °C [38]). There are also plans to adjust retest intervals, according to genus at the ILRI genebank, to one-third of the time predicted for viability to fall to the viability standard, where those predictions are reliable (Y. Woldemariam and J. Hanson, pers. comm.). The revised intervals will be incorporated into the genebank management software used at ILRI.

From personal experience, downloading, sorting, and analysis of genebank monitoring data is not a straightforward exercise, in part because of the length of time over which the data have been collected and entered (or not) into the database—or different databases—by different staff, with a range of associated errors that accrue over time. In future, this process should not constitute a ‘major exercise’; rather, genebank managers should be prepared to ‘regularly’ analyse data or even have some sort of analysis continuously running in the background, within the genebank management software, though the basis of that analysis (algorithms to handle the data, analytical approach) is not known; machine learning may help in this process [43].

### 3.2. Seed Longevity Predictions

Orthodox seeds are not simply defined as those seeds that tolerate desiccation; they are those seeds whose longevity systematically increases in response to a reduction in moisture and temperature [44]. The only longevity model currently available to describe these relations is the Ellis-Roberts viability equations (VEs) [45], which are based on fitting negative cumulative normal distributions to the proportion of viable seeds within samples of seeds stored under different constant moisture and temperature conditions (usually achieved by equilibrating seeds in a particular environment and then sealing them inside air-tight containers, such as aluminium foil bags). The equations have species-specific parameters that quantify the inherent longevity of seeds of a particular species and how longevity changes as moisture content changes. Two further parameters describe the effect of changes in temperature on seed longevity, but the temperature response is thought to be similar across all species [46]. To date, the species-specific parameters have been solved for approximately 70 species, mostly agricultural crops [13]. This means that genebank managers can predict the longevity of seed lots of these species, based on the initial germination test result, for example, using the seed viability constants menu of the Seed Information Database [13]. This is one of the reasons that the initial germination test, ideally made at the time when the seed lot is placed into genebank storage, is so important, not just to confirm the seeds are above the viability threshold, but also to have some indication of how long they might stay above that viability threshold. For example, the predicted length of time it takes for the viability of rice (*Oryza sativa* L.) seeds in long-term storage (6.1% moisture content, −20 °C) to fall to 85% is 255 years when the initial viability is 95%, but 103 years if initial viability is 90% (estimated using the Seed Information Database [13]).

Of course, the VEs are not infallible; there is error associated with any prediction, based on the errors associated with the parameter estimates caused by random (and perhaps experimental) errors in the original data [47], though prediction errors have not usually been calculated. Furthermore, the initial viability result is only an estimate of the true viability of a seed lot [48,49]. Of more concern, the species-specific parameters may not be as robust as previously thought [49,50]. The VEs are also critiqued because the cumulative normal distribution has a particular well-defined shape. In fact, deviances from the basic symmetrical sigmoid curve can be accommodated, and in many cases, attributed to a biological response. For example, viability may be more or less maintained above a threshold value that is less than 100% [51] or there may be a dormancy-breaking response at the start of storage, before viability declines [52]. It would also be possible to describe sub-populations within the seed lot that are responding to storage differently, provided there are sufficient data.

Despite the concerns around using the VEs to make predictions of longevity, they are still extremely useful, not least for emphasising the importance of drying; even seemingly small declines in moisture content, over a certain range, result in significant increases in longevity [27]. The low moisture content limit to this range varies between species, largely owing to differences in seed oil content; it also shifts to a higher moisture content as the storage temperature is reduced [53]. Importantly, drying below this moisture content does not result in either further improvement in longevity or decline in longevity [27,53,54]. As some of the original work in this area found that the low moisture content limit was reached when seeds were equilibrated to a rather low humidity (approximately 10% at 20 °C for seeds subsequently aged at 65 °C) [55], the first official Genebank Standards recommended drying seeds for genebank storage at 10–25 °C and 10–15% RH [56]. Many genebanks installed drying rooms set to these conditions to efficiently dry seeds. Some genebanks check the moisture content of seeds before packing. The official seed testing method to determine seed moisture content is to weigh a sample of seeds before and after oven-drying (at 103 or 130 °C, depending on species [5]). This is a destructive test and may require far more seeds than a genebank would like to use. A more efficient, non-destructive method is to simply check whether the equilibrium relative humidity (eRH) of the seeds is equal or close to the RH of the environment in which they have been dried, using a suitable hygrometer [57]. If they are in equilibrium, the seeds will not be able to dry further, and are ready to be packed; it is not necessary to know the actual moisture content of the seeds.

**Figure 1:**
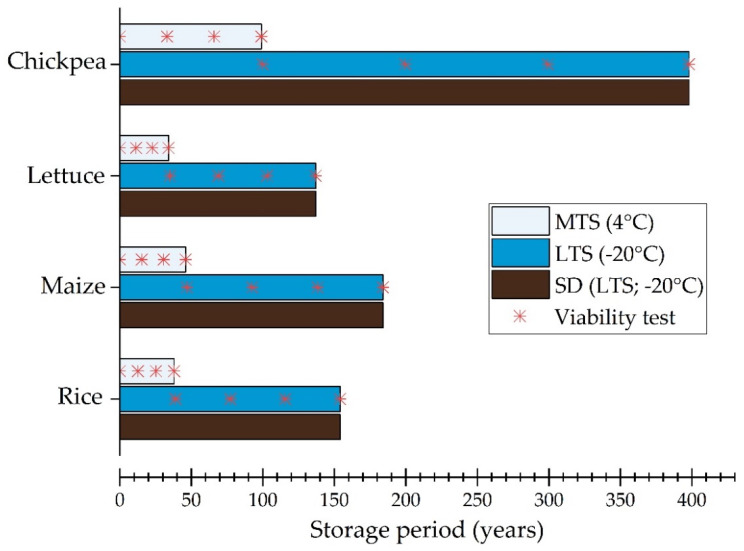
Predicted time for viability to fall from 92% to 85% for seeds of different crops stored in the medium-term store (MTS), the long-term store (LTS), or as a safety duplicate (SD) under LTS conditions in another location. Predications made using the Seed Viability Constants tool of the Seed Information Database [13], based on drying seeds to equilibrium with 15% relative humidity at 15 °C. The red stars indicate the timing of viability monitoring tests, at intervals of one-third of the time predicted for viability to fall to 85%.

The VEs also illustrate why, if different samples of seeds from the same seed lot are stored at the same moisture content (because they are hermetically packed at the same time) at different temperatures, it is not necessary to monitor the viability of both samples from the start. The seeds at the higher temperature will lose viability faster than those at the lower temperature. Thus, for example, it would not be necessary to test the seeds in long-term storage (at the lower temperature) until the viability of the seeds in medium-term storage has declined to the viability standard (Figure 1) [48]. This could save resources if both samples are currently being tested, though of course, it means there would be less viability monitoring data for modelling longevity. Similarly, if samples of seeds are packed for safety duplication (Box 1) in high quality moisture-proof containers at the same time as the samples intended for long-term storage (packed similarly), provided the seeds are stored at the same temperature in the two locations or at a lower temperature in the safety duplicate location than the originating genebank, then it is not necessary to test the seeds that have been sent as the safety duplicate. Moreover, there should not be any real need to include additional samples for retrieval and testing, which would only serve to add extra work load to genebank staff, who may already be challenged to carry out the requisite number of monitoring tests of seeds in the active and base collections.

It is often asked whether moving seeds between temperatures, for example, from the medium-term storage environment to the laboratory, to take a sample for distribution or for viability monitoring, has an effect on the longevity of the seeds. This may happen many times in the ‘lifetime’ of a genebank seed lot. The evidence, again from applying the VEs, is that there is no effect of moving the seeds per se; the viability is affected only by as much as would be expected from the brief period spent at the higher temperature where the seeds are allowed to equilibrate and are sampled [58].

Box 1 **Safety duplication and the Svalbard Global Seed Vault.**

The Genebank Standards [34] recommend, as a safety measure in case of natural disasters, that a sample of all original seeds collected or seed accessions only held by that genebank should be duplicated in another location. Ideally, this location should be in a different country or even continent i.e., somewhere that is not at risk to the same natural and/or human-caused catastrophes, and preferably one where there is no socio-political uncertainty and environmental risk. This “black-box” collection is not active therefore it is the responsibility of the depositor to ensure the sample size is sufficient (enough to carry out at least three regenerations) and of a high quality (>85% germination). If the storage conditions are the same at both locations then the rate of loss in viability of the black-box collection should equal that of the sample in the original genebank, the viability of which is monitored.

The Svalbard Global Seed Vault (SGSV) on Svalbard, Norway, opened in 2008 and offers the long-term storage of safety duplicates from the world’s 1700 national and international genebanks. The vault currently holds more than 1,173,000 samples (www.seedvault.no) and represents the world’s largest collection of crop diversity. It is strategically located in a highly secure zone and is the ultimate insurance policy against loss of plant agrobiodiversity.

### 3.3. Comparative Seed Longevity Studies

A more robust method for assessing the storage potential of different seed lots would be to carry out a seed storage experiment (SSE) rather than just an initial germination test (Table 1). This involves storing samples of seeds at a relatively high temperature and moisture content (e.g., 45 °C after equilibrating seeds at 60% RH and 20 °C) and removing a sample at regular intervals for germination testing [59,60]. After probit analysis of the germination data, the seed lots can be ranked according to the time for viability to decrease to a specific viability [61,62] and/or categorized into longevity categories [63]. If the slopes of the survival curves can be constrained to be the same for all of the seed lots (potentially possible if the seeds are of the same crop), then the theoretical initial viability can be inserted into the VEs to make more precise predictions of longevity. Monitoring intervals for the seeds in genebank storage can then be adjusted on a seed lot basis.

### 3.4. Maturity at Harvest

Seed longevity continues to increase after the seeds have acquired desiccation tolerance, in the late maturation phase of seed development [64,65,66,67]. However, the time when ‘final’ or ‘maximum achievable’ longevity is reached and how long it is maintained thereafter will vary between species, varieties, accessions, and seasons. This is because the longevity of maturing seeds still in the field is highly dependent on both the genotype and the environment, and the interaction of the two. This makes it virtually impossible to consistently harvest different accessions when they have peak longevity.

In the late maturation phase of seed development, the seeds no longer have a vascular connection with the mother plant. The moisture content of the seeds, and thus the availability of water to support different types of chemical reactions, will depend on the ambient conditions (humidity and temperature). The drying that occurs in situ is considered important for stimulating the improvement in seed longevity [52,65], which is why it is often recommended to wait for seeds to dry in the field before harvest [18]. This is easy for shatter-resistant crops; for crops and species that readily disperse their seeds after the vascular connection with the mother plant is broken, delaying the harvest may result in loss of seeds. For such species, bagging inflorescences before they enter the late maturation phase may be necessary [54].

If seeds that have dried on the plant subsequently take up moisture before they have been harvested, depending on the moisture content they attain, longevity may in fact continue to improve, either while the seeds have a high moisture content, provided the seeds do not germinate, and/or upon redrying [68]. If an intermittent moisture content is reached, ageing may occur at a rather fast rate until either the seeds dry again or moisture content increases further. If the moisture content increases to a level where respiration can occur, it is possible that the damage accumulated during ageing will be repaired and seed quality restored/maintained [69]. If the seeds have suffered a substantial amount of damage, they may not able to reach the same longevity as was previously attained. It is ultimately the net changes in seed quality (improvement vs. deterioration) that will determine the potential longevity of the seeds when they are harvested. Developmental events that result in improvements in longevity may also continue ex planta if seeds are harvested prematurely and held at conditions similar to what they might naturally encounter in planta [52,60,70], or upon rehydration if seeds were dried too quickly for maximum quality to be attained [71].

As yet, there is no tool to assess how far seeds have progressed through maturation processes before they are harvested, not least because physical changes that occur during maturation vary so widely between species and varieties [72,73]. In temperate and dry climates, the most reliable method is to see whether seeds are in equilibrium with ambient conditions, by placing a sample of seeds in a portable hygrometer and comparing the seed equilibrium relative humidity with the ambient relative humidity.

### 3.5. Post-Harvest Handling

The post-harvest environment and seed processing operations also affect seed quality and subsequent longevity [61,74]. If seeds have already dried to equilibrium with ambient conditions and reached a relatively low moisture level (<85% equilibrium relative humidity), seeds should be dried as soon as possible to minimize ageing [75]. In semi-arid and arid climates, drying, if necessary, may be done outside under well-ventilated conditions, but avoiding risks such as insect predation or over-heating in direct sunlight. In such environments, care should also be taken not to allow drying to progress too far, as cracking may occur if the seeds need to be threshed. Otherwise, the seeds should be transferred to a controlled drying environment as soon as possible.

As discussed previously, if seeds are harvested with a high moisture content and are likely to be metabolically active, seeds should be dried under conditions that optimally stimulate late maturation phase metabolism. For example, in rice and soybean, the quality of seeds harvested, when still at a high moisture content, was improved by drying for an initial period at a high temperature (45 °C) [50,52,76,77]. It is thought that the loss in moisture is a critical factor controlling the maturation process, by inducing the stress response and other protective mechanisms [78], which significantly increase seed quality. However, drying at such a relatively high temperature is in conflict with the current Genebank Standards that recommend drying mature seeds for long-term storage at a low temperature (5–20 °C) and relative humidity (10–25%) [34]. This recommendation was driven by the requirement for a single, simple, and safe procedure for diverse species in all locations worldwide, and assumed that seeds were already comparatively dry (for example, seeds dried using heated air [79], but requiring further drying to a low moisture content for long-term storage). This was determined by combining the seed viability equation with equations describing the effect of environment on seed drying rate and seed temperature in constant-temperature heated-air dryers in contrasting species [80]. Although the “safe” temperature limit for drying seeds varies between species, high temperatures are usually avoided to reduce the risk of seed deterioration, especially when seeds have a high moisture content and during the later stages of drying when evaporative cooling will no longer suppress the temperature within the seeds [79,80,81]. Despite this, the recommended low temperature and low humidity conditions for post-harvest seed drying are neither species-specific nor dependent upon initial moisture content [34].

### 3.6. Length of Time Before Storage

Most genebanks clean seed lots before packing for storage to remove, for example, off-types, immature, diseased, and damaged seeds. This is often a largely manual process or involves the use of fairly basic equipment such as graded sieves or blowing machines. It is important that the seeds do not sit for too long in an uncontrolled environment while cleaning occurs, as this might mean that they take up moisture and rates of ageing increase. Redrying may be necessary before packing, which should also be done in a controlled environment. Most drying and processing environments, even though they are controlled, are maintained at temperatures that are comfortable for genebank staff, but that are thus higher than desirable from the point of view of maintaining the quality of the seeds. Therefore, it is important that the seeds are packed and transferred to proper genebank storage as soon as possible.

## 4. Statistics of Seed Testing

Genebanks, as a rule, do not conduct experiments on genebank accessions as part of routine operations (Table 1), but they nonetheless do gather data that may be relevant to analyse, for example, to understand the diversity of the material they conserve, to create core-sets based on the diversity represented, or to identify potential gaps in a collection. Some of these data may be suitable for methods of analysis that are typical within the agricultural sciences. However, viability monitoring and other germination data cannot be treated and analysed in the same way [82]. This is because seed germination is a binary response; a seed will either germinate or not germinate, and a sample of seeds will show a germination result that falls between 0% and 100%.

### 4.1. Comparing Two Germination/Viability Results

There are a number of reasons why two germination or viability results might be compared, the most obvious being to compare the results of a germination test with that of a viability test (see above), or the results of a pair of germination tests made using different pre-treatments and/or test conditions. Testing a viability monitoring result against a previous monitoring result or the initial test result would not be common, because genebanks tend to ‘accept’ a viability result and not worry whether or not it is significantly different from any other value. Indeed, genebanks do not even routinely compare a viability monitoring result against the viability threshold value, even though the germination result is only ever an estimate of the whole seed lot viability (because not all the seeds are tested). One test that could be used for comparing two germination results is the χ^2^ test to test whether the probability of success (scored as viable or germinated) is the same for seeds from two different treatments (e.g., stored or not, viability or germination). The algorithm for the χ^2^-test is likely to be based on maximum likelihood estimation, in which case the test is the same as fitting a single-factor binary logistic regression, that is, a generalized linear model (GLM) with logit link function (the link function transforms the response variable, for example, the proportion of germinating seeds, to the linear ‘predictor’ variable that is fitted in the analysis) and binomial error distribution based on the number of seeds tested.

### 4.2. Analysis of a Factorial Germination Experiment

It may be that a dormancy-germination experiment is multi-factorial, in which case the obvious extension to the χ^2^ test is to fit a GLM with different parameters estimating the effect of the different factors included in the experiment. Different link functions may be used to relate the response variable to the predictor [83], but the most common is probably the logit link function, that is, binary logistic regression analysis, for example, [84]. A further extension of this method is to fit a generalized linear mixed model (GLMM) to take into account fixed (factor-related) and random effects (e.g., if seeds are sown across multiple test units such as Petri dishes or rolled paper towels) [82,85]. It is rarely appropriate to use analysis of variance (ANOVA), because the nature of germination data is likely to violate the assumptions of the analysis; that is, that the errors after fitting the model follow the same, normal distribution across treatment groups [82].

### 4.3. Analysing a Series of Germination Results

Although not routine in most genebanks, recording the progress of germination of a sample of seeds in a germination test over some days or weeks (or in some cases, even longer) is common in studies related to determining optimum dormancy-breaking/germination requirements and understanding seed vigour (speed of germination). Germination progress data are also collected to assess seed lot behaviour in response to stress (e.g., water stress) or, conversely, to stimulants, for example, [86,87]. By convention, many analyses of such behaviour have neither taken the binomial error distribution of germination behaviour into account nor used independent samples, e.g., [88]. If independent samples are used, for scoring just once and then discarding, again, a GLM or GLMM might be used to analyse the data; if samples are repeatedly scored for germination, time-to-event model fitting would be more appropriate [83]. Germination data for a series of samples from a seed lot in storage can be analysed by fitting a GLM. By convention, this has been by probit analysis (i.e., fitting a GLM with a probit link function) and is the basis of the Ellis-Roberts viability equations (see above).

## 5. Concluding Remarks

Genebank managers often come from diverse backgrounds, with different scientific training and experience. On the other hand, to be a genebank manager requires a diverse set of skills and acquaintance with, if not knowledge of, a broad range of topics. As such, it can be difficult to find an ideal person to fill a senior genebank management position, unless there is an applicant who is moving from another genebank or has been an understudy for the role. Consequently, it may be likely, or even inevitable, that some expertise gets overlooked and/or is difficult to find compared with a few decades ago, when for example, plant taxonomy and seed science were perhaps more likely to be covered in botany and plant science degrees. As such, incoming genebank managers may be on a steep learning curve and be limited by a lack of understanding of some aspect(s) of genebank science. It is key to the management of a genebank that staff understand the reasons behind patterns of germination shown by individual crops and seed lots; are primed to make efforts to increase the efficiency of operations; and are able to communicate this to donors, reviewers, and the public. If not the head of the genebank, then other genebank staff should have training in seed science and technology and/or access to seed physiology experts, and deliberate strategic investments should be made in capacity building in this area.

Seed handling has been a focus of recent quality management audits and external reviews that have been conducted under the CGIAR Genebank Platform [89]. Standard operating procedures (SOPs) have been drafted and audited for key operations at each of the 11 CGIAR genebanks. In addition to document audits, expert reviewers have visited each genebank to see the procedures in practice and to validate individual SOPs. A large number of the reviewers’ observations and recommendations concerned seed processing, handling, and multiplication of seed lots and related data management. It is clear that seed quality management practice is a highly dynamic and influential aspect of genebank management, where a lack of oversight at any point in the long history of a permanent genebank can profoundly impact efficiency and long-term conservation. Continual expert input and review, and research and experimentation, as well as innovation and automation on various areas of seed management, clearly play major roles in the sustainability of major seed collections.

In this review, we have discussed various aspects of seed physiology that are particularly relevant for seed genebanks if appropriate decisions about the handling of seed germplasm are to be made. Some of this may seem like common sense to those that are trained in seed science, but in fact, many of the observations relate to questions that do get asked by genebank staff at all management levels. Further, there should be more interaction between the seed science/testing community and genebanks. This would help genebank staff stay abreast of new scientific developments in seed physiology and, in particular, testing methods, and thereby help them to continue to improve the efficiency and effectiveness of genebanks operations. It is also important for seed scientists to be aware of areas on which to focus their research to support the work of the genebanks in conserving and making available plant genetic resources.

## Figures and Tables

**Table 1:** Examples of how genebank operations might differ if resources are limited and there is a need for high levels of efficiency compared with what could be done if resources are available, which would be more sound and/or beneficial from a scientific perspective.

Optimum Strategy from a Genebank Management Perspective	More Scientifically Sound or of Interest from a Scientific Perspective
Only one sample of each accession in the active and base collections.	To compare the physiological response of seed lots produced in different crop seasons or environments; to have seeds of different ages to test at the same time (e.g., to understand seed longevity).
Once-over harvesting strategy.	Harvest seeds as they reach maturity.
Stop monitoring seeds once the seed lot that represents an accession has been replaced.	Continue monitoring viability to collect more data to inform seed longevity. This is better, not just because there are more data, but also because more of the data will cover the range where viability is expected to decline faster, enabling more robust model fitting.
Only consider one or a few different dormancy-breaking treatments at a time.	Factorial dormancy breaking/germination experiment, with different treatments and/or germination temperatures and treatment combinations. This should be a priority for ‘new’ species where there is little information on dormancy and germination requirements.
Initial viability test to confirm initial seed quality is sufficient.	Initial seed storage experiment to estimate initial seed storage potential, for setting seed lot-based monitoring intervals, and/or for confirming that the ranking of seed lots for longevity based on experimental storage corresponds with the ranking in genebank storage.
Minimal viability monitoring tests, e.g., only test a subset from each harvest season.	Monitor the viability of all samples at frequent intervals to get more data on relative seed longevity of different samples and of the same samples in different storage environments (e.g., medium- vs. long-term storage).
Only score for germination once or twice during a viability monitoring test.	Regular scoring of germination during a germination viability monitoring test to get information on speed of germination (vigour measures) and how vigour declines as seeds age.
